# Response and Flow Characteristics of an Angular Momentum Flowmeter

**DOI:** 10.3390/s25216728

**Published:** 2025-11-03

**Authors:** Hao Zan, Qiusheng Jia, Chengli Liu, Jiabao Liu, Fuji Huang, Shenmei Zhou

**Affiliations:** 1 Ningbo Institute, Northwestern Polytechnical University, Ningbo 315103, China; 2 School of Energy and Power Engineering, Jiangsu University of Science and Technology, Zhenjiang 212100, China; 3 Beijing Power Machinery Institute, Beijing 100074, China; jia_qiusheng@126.com; 4 The 49th Research Institute of China Electronics Technology Group Corporation, Harbin 150028, China; xudong_1114@126.com; 5 Jiangsu Aerospace Measurement and Control Technology Co., Ltd., Zhenjiang 212000, China; liujiabao2024@163.com (J.L.); zhoushenmei@jsktck.com (S.Z.)

**Keywords:** angular momentum flowmeter, visualization experiment, dynamic response, numerical simulation

## Abstract

The angular momentum flowmeter addresses critical challenges in aviation fuel flow measurement during commercial flight operations. This study designed a visualization platform to observe the dynamic responses of internal components under varying flow conditions. By employing the sliding mesh method coupled with an angular momentum algorithm, it enabled the dynamic rotation simulation of the upstream straight-bladed rotor and provided calculation of the deflection angle in the downstream straight-bladed rotor of an angular momentum flowmeter. Experimental results categorize the flow process into three distinct regimes based on flat and spiral spring response states: pre-spring, single-spring, and dual-spring regimes. Under a flow condition of 0.091 kg/s, the upstream straight-bladed rotor maintained stable rotation at a speed of 1.1 rad/s. At a flow rate of 0.20 kg/s, the flat spring initiated outward expansion, and with further increase in flow rate, the rotational speed of the upstream straight-bladed rotor remained within the range of 25.34–26.21 rad/s. Mathematical analysis demonstrates that the flat spring configuration extends the lower measurement limit and promotes dissipation of the secondary vortex through dominant kinetic energy of the primary vortex during dual-spring operation, thereby improving high-pressure zone stability. This work elucidates the operational mechanism of angular momentum flowmeters and provides a theoretical basis for structural optimization.

## 1. Introduction

As the power core of an aircraft, the performance parameters of an aero-engine directly determine its safe operation, energy efficiency, and operational stability. Fuel flow has always been a critical parameter, closely related to the performance of aero-engines, and research on fuel flow measurement techniques has never ceased [1,2]. In the complex and variable high-altitude environment, the precise measurement of fuel flow is not only key to optimizing engine control strategies but also serves as an important technical foundation for improving the thermodynamic efficiency of the combustion chamber and extending flight endurance [3]. It is also closely associated with the safety and reliability of aircraft under various flight conditions [4] and is regulated in real-time as a control object [5].

The mass flow measurement techniques currently employed in the aviation industry are primarily categorized into two systems: Indirect measurement methods estimate mass flow by coupling multiple parameters, with representative technologies including turbine-density meter combined systems [6,7,8,9], temperature- and pressure-compensated orifice plate differential pressure devices [10,11,12,13], and ultrasonic flowmeters utilizing acoustic modeling [14,15,16,17]. Direct measurement methods, in contrast, determine mass flow directly based on fundamental principles of fluid dynamics. These include instruments leveraging the Coriolis effect [18,19,20,21], thermal-conduction-based flow sensors [22,23,24], and angular momentum flowmeters operating on the conservation of momentum. These approaches differ significantly in measurement accuracy, environmental adaptability, and practical engineering feasibility.

The extreme operating conditions encountered by modern aero-engines impose stringent technical requirements on flow measurement devices [25]. Within a severe temperature variation range from −55 °C to 300 °C, the physical properties of fuel (such as viscosity and density) undergo nonlinear variations and may even experience chemical evolution, including hydrocarbon cracking. These changes significantly degrade the accuracy of indirect measurement methods that rely on property compensation [26]. Meanwhile, persistent vibration at the 20 g level and transient shock loads create a highly complex dynamic environment, making it difficult for traditional differential-pressure and volumetric flowmeters to maintain stable operation. Although Coriolis mass flowmeters offer the advantage of direct mass flow measurement, their vibration-sensitive operating principle makes them susceptible to external excitation, leading to issues such as mechanical resonance and signal drift. As a result, their operational reliability remains limited in aeronautical dynamic applications.

The innovative design of the angular momentum flowmeter offers a new pathway to address the aforementioned technical challenges. This device employs a dual-rotor and spring torque system to achieve direct mass flow reading by accurately measuring changes in the angular momentum transferred by the fluid medium.

## 2. Experimental Schemes and Mathematical Models

### 2.1. Structure of the Angular Momentum Flowmeter

**Figure 1:**
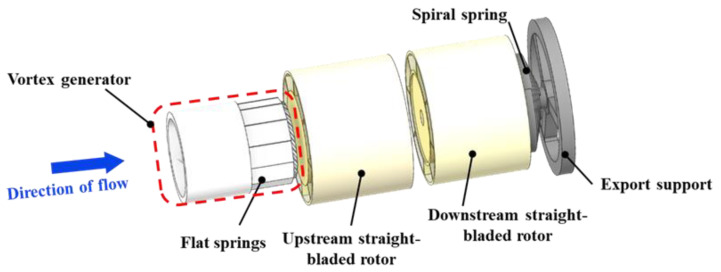
Enlarged view of the angular moment flowmeter.

The internal structure of the angular momentum flowmeter is illustrated by the exploded view in Figure 1. Key components arranged sequentially along the flow path include: an inlet nozzle that guides and accelerates the fluid; a vortex generator responsible for creating specific rotational motion; an upstream straight-bladed rotor interacting with the incoming swirling flow; a downstream straight-bladed rotor; and a helical spring measuring the torque transmitted from the downstream rotor.

### 2.2. Experimental Schemes

**Figure 2:**
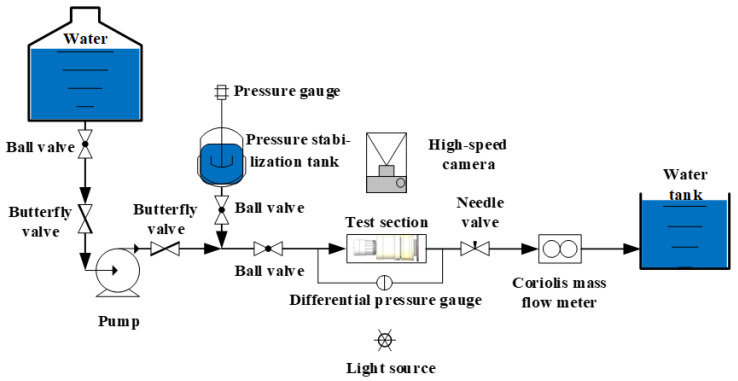
Visualization experiment for the angular moment flowmeter.

As shown in Figure 2, to visually observe the dynamic response of internal components within the angular momentum flowmeter, the test section of the visualization platform was made fully transparent. High-speed cameras were employed to capture the dynamic response information of the vortex generator, upstream straight-blade rotor, and downstream straight-blade rotor. Water was supplied from a vertical water tank. The tank outlet was connected to a centrifugal pump. A pressure stabilization system was installed in parallel at the centrifugal pump outlet. This stabilization system primarily comprised a pressure stabilization tank, a pressure gauge, and a ball valve, ensuring stable pressure at the centrifugal pump discharge. The centrifugal pump outlet was connected to the visualization test section via a straight pipe segment. Following the test section, a standard flowmeter was connected. This standard flowmeter was a Coriolis mass flowmeter.

**Figure 3:**
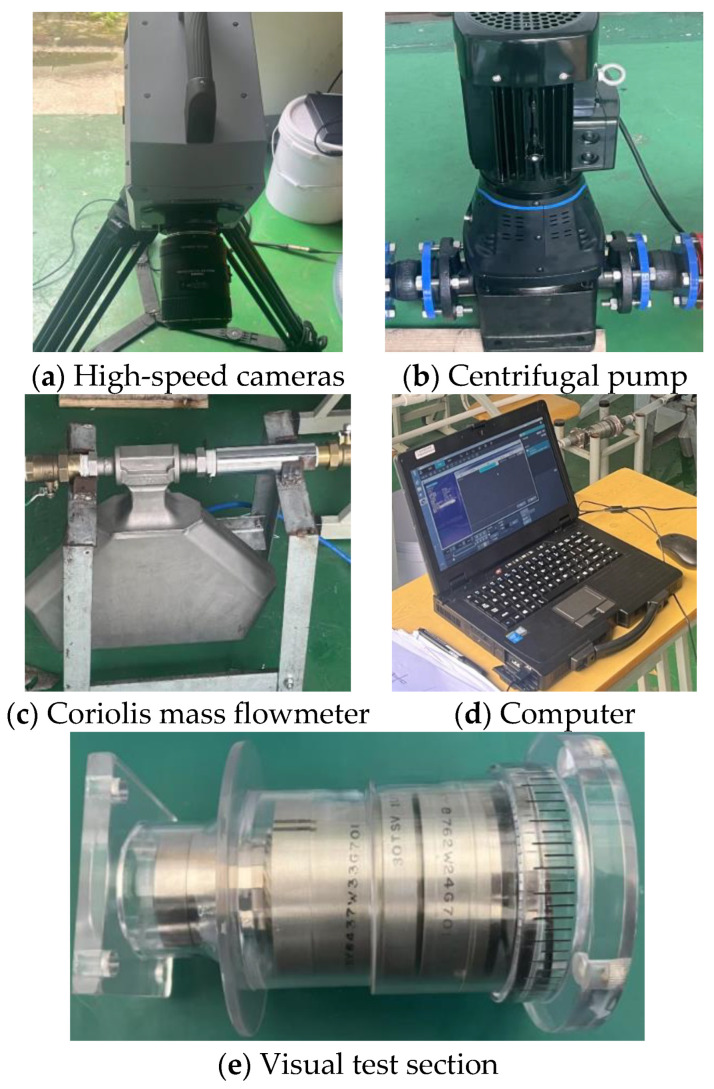
Component diagram of the experimental system.

The present study fabricated a flow visualization platform using stereolithography (SLA) additive manufacturing technology. The core measurement components were precisely assembled and integrated within an optical observation chamber. A key feature of this chamber is its fully transparent design, ensuring global visibility of the flow field. A calibration grid was incorporated in the transition section at the flow channel outlet to quantitatively capture the deflection displacement of the downstream straight-bladed rotor, as shown in Figure 3. High-speed imaging was employed to observe the rotational speed of the upstream straight-bladed rotor and the deflection angle of the downstream rotor. A FASTCAM SA-Z high-speed camera, equipped with a SIGMA MACRO 105 mm F2.8 EX DG OS HSM lens, was used, capable of achieving a maximum recording speed of 20,000 frames per second (fps) at a resolution of 1024 × 1024 pixels. The correlation between rotor speed and image frame count was established. Once the frames per second (FPS) setting of the high-speed camera was determined, the rotational speed could be converted into the number of images required per revolution. A recording frequency of 250 FPS was adopted, and the position of the downstream rotor was determined using a signal blade installed on its outer hub. Under steady flow conditions, the deflection angles of both the flat spring and the downstream rotor were measured. Flow rate calibration was performed using a Coriolis mass flowmeter with a flow range of 0–1.2 kg/s, a measurement accuracy of ±0.2%, a nominal diameter of DN20, a maximum pressure loss of 2 MPa, and an operating temperature range of −20 to +150 °C.

Under initial conditions, the butterfly valve and ball valve at the centrifugal pump inlet were opened. Utilizing the high liquid level in the water tank, gas was purged from the pump via its vent port until the centrifugal pump casing was completely filled with water. Subsequently, the downstream butterfly valve was opened, the centrifugal pump was started, and the ball valves on the stabilization tank and downstream of the pump were opened. The dynamic behavior of the angular momentum flowmeter was then observed.

The dynamic responses of individual components, obtained through visualization experiments, served as the basis for subsequent numerical simulations and mathematical calculations conducted in this study. To analyze the flow characteristics within the channel, we calculated the average fluid velocity (*u*). This velocity was derived from the volumetric flow rate (*Q*) divided by the constant cross-sectional area (*A*) of the inlet channel, according to *u* = *Q*/*A*. The volumetric flow rate *Q* was measured by the aforementioned Coriolis mass flowmeter, which has an accuracy of 0.2%, and was converted from mass flow rate using the fluid density.

### 2.3. Mathematical Model

The upstream straight-blade rotor constitutes the core component of the angular momentum flowmeter. Governed by the fluid driving torque (*Td*), the rotor initiates rotation while simultaneously being subjected to the following counteracting torques: the viscous drag torque from the rotor hub (*Th*), the viscous drag torque at the rotor end faces (*Tw*), the magnetic reluctance torque (*Tm*), and the bearing friction torque (*Tb*).

(1) $$J\frac{dw}{dt}=T_{d}-T_{h}-T_{w}-T_{m}-T_{b},$$

When the rotor angular velocity stabilizes, dw/dt=0, yielding:

(2) $$T_{d}-T_{h}-T_{w}-T_{m}-T_{b}=0$$

## 3. Numerical Modeling

### 3.1. Computational Model and Grid

The geometric model was constructed based on the actual shaft configuration of the angular momentum flowmeter, with explicit incorporation of assembly clearances between the upstream straight-blade rotor, vortex generator, and downstream straight-blade rotor. To reduce computational expense, the shaft and bearings were excluded during fluid domain extraction. The integrated flow path comprises five functional sections: Inlet support, vortex generator (incorporating swirl vanes and flat springs), upstream straight-blade rotor, downstream straight-blade rotor, and outlet support.

**Figure 4:**
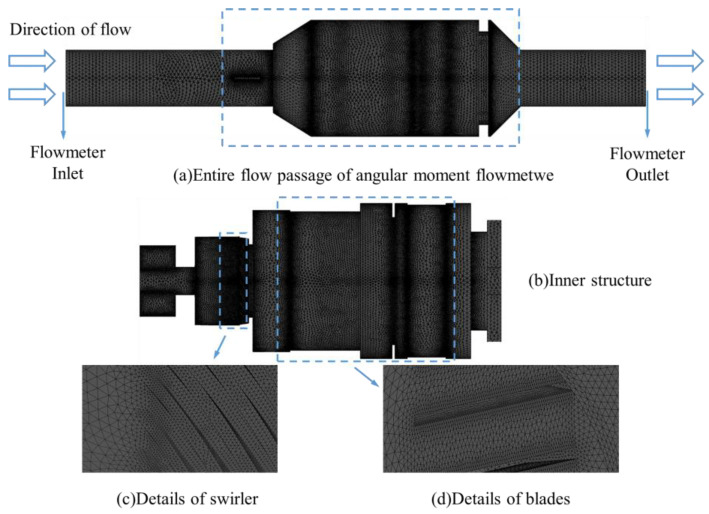
Computational grids of the angular moment flowmeter.

As shown in Figure 4, given the passively rotating upstream straight-blade rotor requiring enhanced mesh adaptability, an unstructured grid topology was implemented despite its higher computational demand. Critical regions exhibiting large curvature gradients, narrow clearances, or abrupt geometric transitions underwent localized grid refinement to preserve flow field accuracy. Mesh accuracy exerts a direct impact on the fidelity of fluid flow simulations. In this study, the entire flow passage of the angular momentum flowmeter was discretized using Fluent Mesh, with the orthogonal quality of all computational grids exceeding 0.15.

### 3.2. Turbulence Model

All natural flows adhere to fundamental physical principles, namely the three governing laws of fluid mechanics. In the experimental campaign, ambient-temperature water served as the working fluid. For numerical simulations, the fluid was treated as incompressible. With no heat transfer or temperature variation occurring during the computational process, the energy equation was excluded from consideration. Consequently, only the conservation laws of mass (continuity equation) and momentum were solved. Within an orthogonal coordinate system, the governing equations thus take the following form [27]:

The conservation of mass equation is as follows:

(3) $$\frac{\partial u_{\mathrm{i}}}{\partial x_{i}}=0$$

The momentum conservation equation is as follows:

(4) $$\frac{\partial u_{\mathrm{i}}}{\partial x_{i}}+\frac{u_{j}\left(\partial u_{i}\right)}{\partial t}+u_{j}\frac{\partial u_{i}}{\partial x_{j}}=-\frac{1}{\rho }\frac{\partial P}{\partial x_{i}}+v\frac{\partial^{2}u_{i}}{\partial x_{i}\partial x_{j}}$$

In the equation, *x_j_* and *u_j_* are the velocity components in the Cartesian coordinate system and its coordinate system, *t* is time, *ρ* is fluid density, *p* is pressure, and *v* is fluid viscosity.

For turbulence model selection, accurate resolution of near-wall flow behavior was prioritized. The Shear Stress Transport (SST) *k-ω* model proposed by Menter [28] was adopted, which synergistically combines the strengths of: The *k-ω* formulation’s superior accuracy in near-wall regions and the *k*-*ε* model’s reduced sensitivity to inlet flow parameters. The dimensionless governing equations of this model are expressed as follows:

The eddy viscosity equation is as follows:

(5) $$V_{T}=\frac{a_{1}k}{\max\left(a_{1}w_{1}S\;F_{2}\right)}$$

The turbulent kinetic energy *k* equation is as follows:

(6) $$\frac{\partial \left(\rho k\right)}{\partial t}+\frac{\partial }{\partial x_{j}}\left(\rho U_{j}k\right)=P_{k}+\frac{\partial }{\partial x_{j}}\left[\left(\mu +\frac{\mu_{t}}{\sigma_{k3}}\right)\frac{\partial k}{\partial x_{j}}\right]-\beta^{\prime }k\omega$$

The turbulence frequency *ω* equation is as follows:

(7) $$\frac{\partial \left(\rho \omega \right)}{\partial t}+\frac{\partial }{\partial x_{j}}\left[\rho U_{j}\omega -\left(\mu +\frac{\mu_{t}}{\sigma_{\omega 3}}\right)\right]\;\;=a_{3}\frac{\omega }{k}P_{k}-\beta_{3}\rho \omega^{3}+2\left(1-F_{1}\right)\rho \frac{1}{\omega \sigma_{\omega 3}}\frac{\partial k}{\partial x_{i}}\;\frac{\partial \omega }{\partial x_{i}}$$

The equations for the mixture functions *F*_1_ and *F*_2_ are as follows:

(8) $$arg_{1}=min(\max\left(\frac{\sqrt{k}}{\beta^{\prime }wy},\frac{500v}{y^{2}w}\right),\frac{4\rho k}{{CD}_{km\sigma_{w2}}y^{2}})$$

(9) $$F_{1}=tanh(arg_{1}^{4})$$

(10) $$arg_{2}=\max\left(\frac{\sqrt{k}}{\beta^{\prime }wy},\frac{500v}{y^{2}w}\right)$$

(11) $$F_{2}=tanh(arg_{2}^{2})$$

(12) $$CD_{kw}=max(2\rho \frac{1}{\sigma_{w2}}\nabla k\nabla w,1.0\times 10)$$

where *S* is the invariant measure of the strain rate. The mixture function *F*_1_ takes a value of 1 in the near-wall region and 0 away from the wall, thereby enabling a smooth transition between the *k-ω* and *k-ε* turbulence models. *α*_1_, *σ_k_*_3_, *β*′, *σ_w_*_2_, *σ_w_*_3_, *α*_3_, and *β*_3_ are constants.

### 3.3. Numerical Methods and Boundary Conditions

Numerical simulations were performed using the commercial CFD software Ansys Fluent (2022 R1 release). The SST *k-ω* turbulence model was selected for computational modeling. Considering the operational characteristics of the angular momentum flowmeter, a mass-flow-inlet boundary condition was imposed at the domain inlet, while a pressure-outlet condition (with reference pressure set to atmospheric) was specified at the outlet. Interface conditions were defined between rotating and stationary zones. Pressure-velocity coupling was resolved via the SIMPLEC algorithm, with second-order discretization schemes applied to all remaining transport variables.

Phase 1 was steady-state initialization. The governing equations of motion were not applied to the upstream straight-blade rotor during this phase. Simulations were executed under prescribed inlet mass flow rates until all residuals reached 1 × 10^−5^, at which point solutions were considered converged. Phase 2 was transient simulation. The converged steady-state solution subsequently served as the initial field. User-Defined Functions (UDFs) were dynamically linked to introduce rotor dynamics for unsteady computations of the upstream straight-blade rotor.

This study involves fluid simulation of rotating structures. For such simulations, Fluent provides users with three primary methods. As shown in Table 1, the first is the Moving Reference Frame (MRF) approach, which is a steady-state approximation. It cannot capture dynamic changes during motion and only provides averaged flow field results. The second is the Dynamic Mesh method, known for its high flexibility. It can simulate complex deformations, fluid–structure interactions (such as piston motion and flapping-wing aircraft), and processes involving large displacements. However, it requires frequent mesh regeneration during computation, which can easily lead to numerical divergence and demands excessive computational resources and time. The third is the Sliding Mesh technique, which can capture transient flow field information and is suitable for complex motions. Additionally, it does not require mesh regeneration and demonstrates favorable numerical convergence.

After comprehensively comparing the simulation approaches for the three rotational structures, the sliding mesh method is adopted in this study. By invoking motion functions via User-Defined Functions (UDF) in Fluent, the upstream straight-bladed rotor achieves rotational states transitioning from stationary to startup, acceleration, and finally stabilization. The deflection angles of the downstream straight-bladed rotor under different flow rates are thereby obtained.

**Figure 5:**
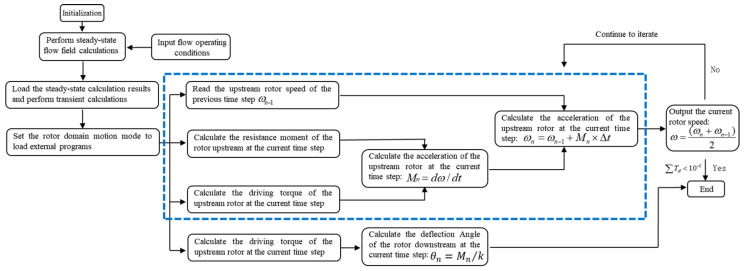
The calculation process of the UDF program method.

The overall simulation process, as illustrated in Figure 5, utilizes UDF programming to implement dynamic simulation of the upstream rotor’s free rotation under fluid impact and to extract numerical values of the deflection angles of the downstream rotor subjected to fluid impact. The specific procedure is as follows: based on simulation results from the previous time step, the driving torque and viscous resistance torque acting on the rotor are determined, yielding the rotational speed of the upstream straight-bladed rotor at that prior step. Subsequently, using results from the current time step, the acceleration of the upstream rotor and the driving torque on the downstream rotor are calculated. This process ultimately provides the rotational speed of the upstream straight-bladed rotor and the deflection angle of the downstream straight-bladed rotor for the current time step.

### 3.4. Grid Independence Test

The flat spring within the angular momentum flowmeter was fully deployed at the operating condition of 0.4 kg/s mass flow rate, with stabilized internal flow field development. Given the high simulation accuracy achieved at this condition, it was selected as the validation flow point for the grid independence study.

Five distinct mesh configurations were generated for the fluid domain of the angular momentum flowmeter, with cell counts of 3.47 million, 4.78 million, 5.25 million, 6.89 million, and 7.92 million respectively. These mesh systems are cataloged in Table 2.

**Figure 6:**
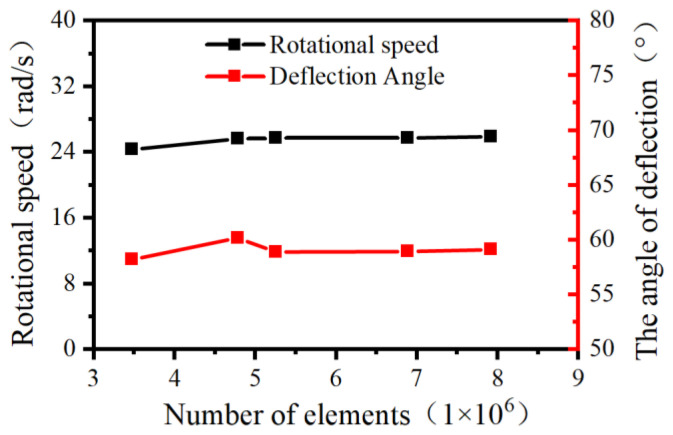
Grid independence verification of the angular momentum flowmeter.

As evidenced in Figure 6, both the rotational speed of the upstream straight-blade rotor and the deflection angle of the downstream straight-blade rotor exhibit bounded fluctuations. Beyond the mesh refinement level of 5.25 million cells, these parameters stabilize within a variation threshold of 1%. Consequently, the computational model with 5.25 million cells was selected for subsequent simulations.

## 4. Results and Discussion

### 4.1. Response of the Angular Moment Flowmeter

**Figure 7:**
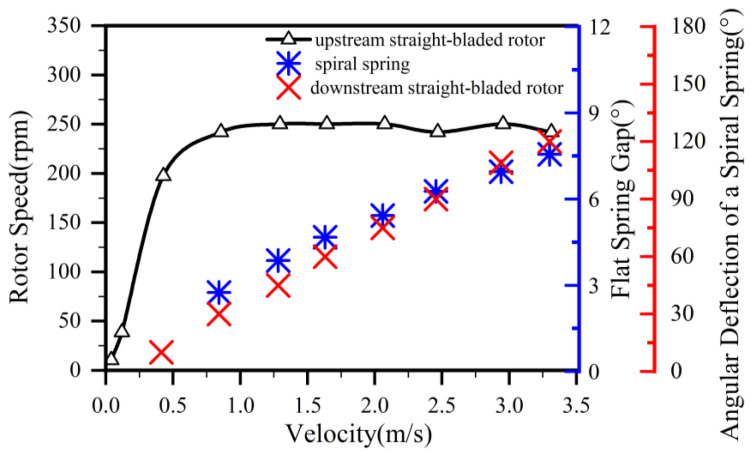
Dynamic response curve of the angular momentum flowmeter.

Visualization experiments were conducted to characterize the response of key components within the angular momentum flowmeter across varying flow rates. As shown in Figure 7 and Table 3, structural responses were observed at mass flow rates ranging from 0.05 to 0.81 kg/s on the visualization platform. Experimental imaging confirmed that all incoming fluid entered the vortex generator. The upstream straight-blade rotor initiated rotation at a flow velocity of 0.0405 m/s, The spiral spring coupled to the downstream rotor exhibited a response at 0.428 m/s, with a deflection angle of 10°.

With increasing flow rate, both the rotational speed of the upstream rotor and the deflection angle of the downstream rotor progressively increased. This trend continued until flow through the helical channels of the vortex generator forced the flat spring to deflect outward at a critical velocity of 0.856 m/s. As demonstrated in Figure 7, further flow augmentation resulted in greater outward deflection of the vortex generator’s flat spring while rotor rotational speeds maintained stability beyond this threshold.

The entirety of fluid entering the angular momentum flowmeter was directed into the vortex generator’s flow passages. At lower flow rates, the upstream straight-blade rotor initiated rotation while the downstream straight-blade rotor underwent angular deflection. With progressive flow augmentation, the flat spring within the vortex generator ultimately deflected outward. Beyond this threshold, the rotational speed of the upstream rotor maintained relative stability. Three distinct operational regimes were identified: Pre-spring response regime, single-spring response regime, and dual-spring response regime.

**Figure 8:**
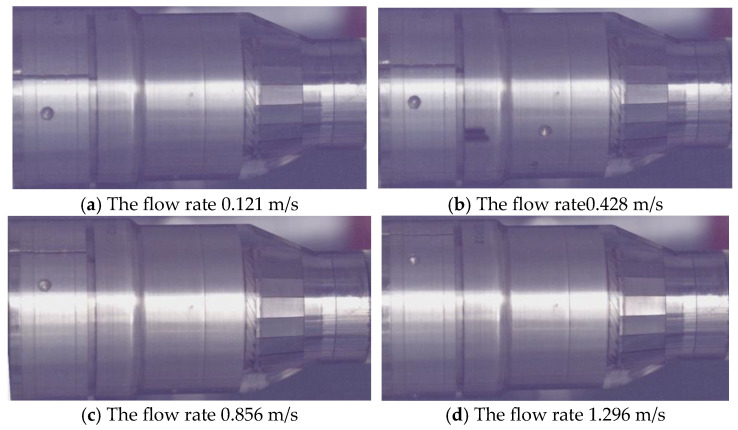
Visual response of the internal components of the angular momentum flowmeter.

Figure 8 illustrates the visual responses of internal components within the angular momentum flowmeter. In Figure 8a, the flat spring of the swirl generator remains inactive, the upstream straight-blade rotor rotates uniformly, and the downstream straight-blade rotor is at the critical point of deflection; in Figure 8b, the flat spring of the swirl generator remains inactive, the upstream straight-blade rotor rotates uniformly, and the downstream straight-blade rotor is deflected; in Figure 8c, the flat spring of the swirl generator is activated, the upstream straight-blade rotor rotates uniformly, and the downstream straight-blade rotor is deflected; in Figure 8d, the flat spring of the swirl generator is activated, the upstream straight-blade rotor rotates uniformly, and the downstream straight-blade rotor exhibits a larger deflection angle.

### 4.2. Comparative Analysis of Three Distinct Operational Regimes

**Figure 9:**
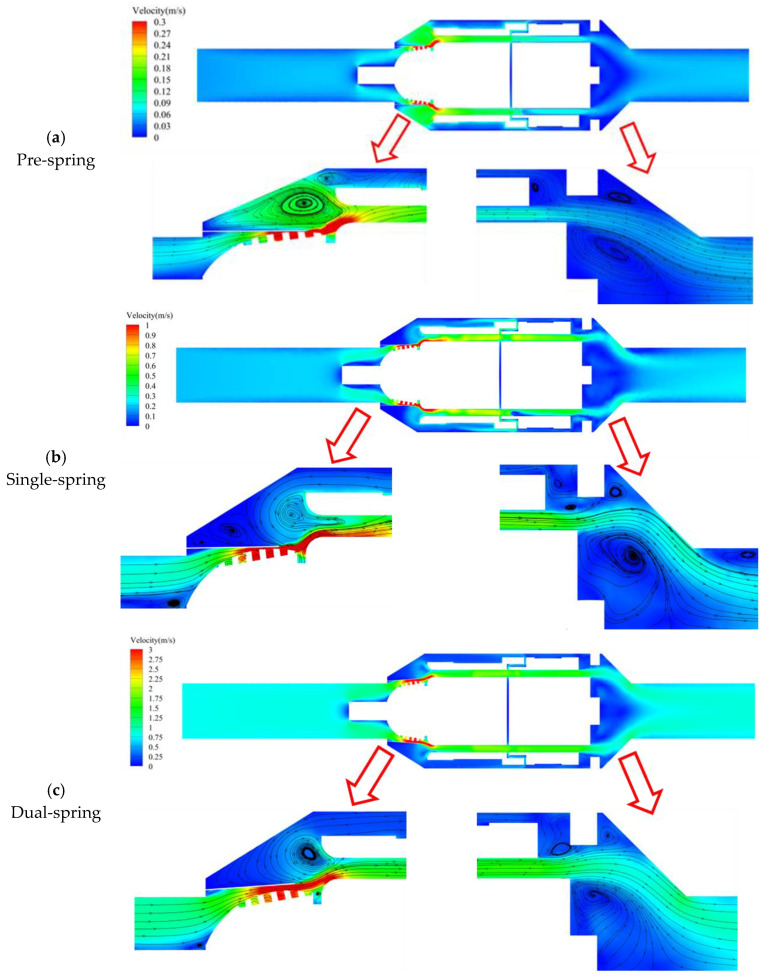
Distribution of streamlines in the full flow passage of the angular momentum flowmeter at the cross-section x = 0 mm.

As illustrated in Figure 9, three response regimes were quantified at distinct mass flow rates: Pre-spring response regime: 0.029 kg/s, single-spring response regime: 0.104 kg/s, and dual-spring response regime: 0.504 kg/s. Within the inlet flow domain of the angular momentum flowmeter, streamline distributions exhibit high uniformity. Upon entering the inlet support structure, fluid experiences flow obstruction, subsequently impinging on the support nose and diverting radially outward. The reduced cross-sectional area in the inlet support section accelerates flow velocity. As fluid progresses downstream to the vortex generator assembly, further velocity amplification occurs, reaching maximum magnitude at the generator’s trailing edge. This high-velocity flow impinges on the upstream straight-blade rotor with significant angular momentum, inducing rotor rotation. Flow velocity progressively attenuates through the inter-rotor passage between upstream and downstream straight-blade rotors, ultimately exiting through the outlet support structure.

As depicted in Figure 9a, the maximum flow velocity occurs at the outlet of the vortex generator’s flat spring assembly within the angular momentum flowmeter, primarily due to extreme flow constriction at this minimal passage diameter where throttling effects create a high-velocity zone, giving rise to vortex formation between the flat spring and housing; whereas both pre-spring and single-spring response regimes exhibit two vortex regions, the dual-spring regime features only one. Comparative analysis (Figure 9a,b) reveals consistent spatial positioning of the primary vortex across regimes, but differential secondary vortex positioning: the pre-spring regime locates it in the upper-right quadrant relative to the primary vortex, whereas the single-spring regime positions it in the lower-left quadrant—presumably displaced by accelerated flow within the primary vortex. In the dual-spring regime (Figure 9c), a singular vortex persists at the primary vortex location observed in other regimes, with the secondary vortex presumably dissipated by excessive momentum in the primary vortex.

As fluid enters the upstream straight-blade rotor domain, the high-velocity zone extends correspondingly, though flow velocity progressively attenuates throughout the inter-rotor passage; at the outlet support structure, flow separation phenomena emerge due to the divergent flow passage geometry, forming symmetrical vortices upon exit. Concurrently, increasing flow velocity elevates pressure along the streamwise direction, inducing central vortex formation within the flow core. As shown in Figure 9a, only a single vortex is observable above the main flow at the outlet during the pre-spring regime under baseline velocity conditions. With increasing flow velocity in the single-spring regime (Figure 9b), three distinct vortices become clearly discernible above the primary flow. Further velocity augmentation in the dual-spring regime (Figure 9c) reveals two persistent vortices accompanied by one nearly dissipated vortex above the main flow trajectory.

### 4.3. Comparative Analysis of Angular Moment Flowmeter

**Figure 10:**
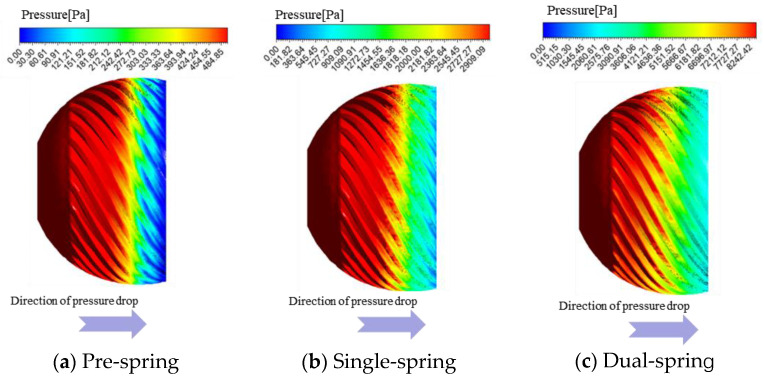
Wall pressure cloud map of the three response stages of the cyclone.

As shown in Figure 10, the surface pressure contours of the cyclone separator are presented for three distinct stages, with panels (a), (b), and (c) corresponding to the Pre-spring, Single-spring, and Dual-spring stages, respectively. The vortex generator serves as a critical metrological component within the angular momentum flowmeter, exhibiting distinct wall pressure distributions across all three response regimes where the demarcation between high- and low-pressure zones consistently aligns with the trailing edge of the flat spring. Upon entering the vortex generator assembly, fluid initially impinges on the arcuate surface, experiencing rapid acceleration through the subsequent converging section; this velocity amplification intensifies impact forces on the generator surface, resulting in uniform pressure distribution along the curved profile. Constrained by the flat spring, fluid is directed exclusively into the helical passage between the spring and generator wall. Downstream of the spring’s trailing edge, abrupt flow expansion occurs, establishing a definitive pressure dichotomy along the spiral channel walls with the spring’s terminus acting as the demarcation line between high- and low-pressure regions.

Across all three response regimes, the vortex generator exhibits consistent wall pressure distribution trends with high- and low-pressure zones consistently demarcated at the flat spring’s trailing edge; the maximum wall pressure magnitudes progressively increase from 484.85 Pa in the pre-spring regime to 2909.09 Pa in the single-spring regime, culminating at 8242.42 Pa in the dual-spring regime.

**Figure 11:**
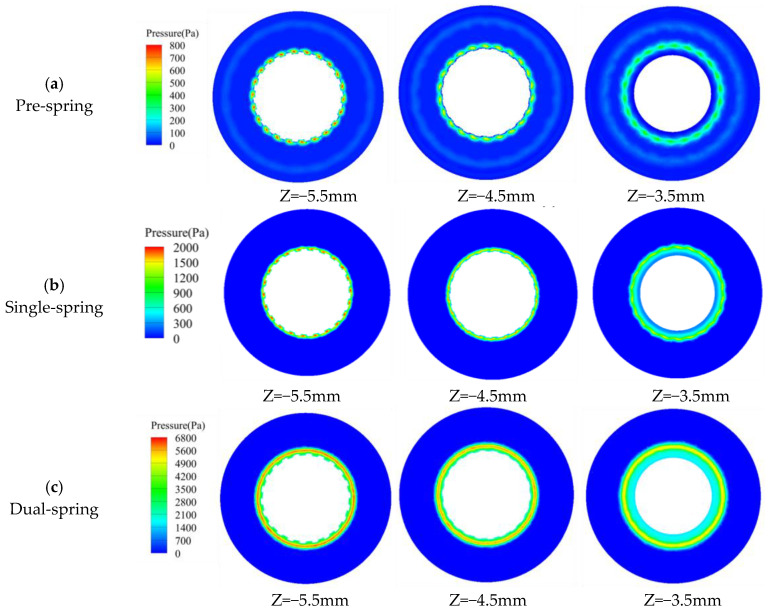
Internal structure slice pressure cloud map of the angular momentum flowmeter.

Figure 11 presents pressure contour plots across three transverse sections of the vortex generator and upstream straight-blade rotor assembly, with Z = −5.5 mm representing the section proximal to the vortex generator and Z = −3.5 mm positioned near the upstream rotor. In the pre-spring regime (Figure 11a), the high-pressure core localizes at the central flow passage of the vortex generator; progressing toward the upstream rotor (Z = −3.5 mm), pressure magnitude progressively diminishes while the high-pressure zone radially expands. The single-spring regime (Figure 11b) maintains spatial consistency of the high-pressure core within the generator’s passage, though pressure intensity moderates and the core radially diffuses when transitioning toward the rotor plane. Conversely, the dual-spring regime (Figure 11c) exhibits high-pressure concentration adjacent to the deployed flat spring due to its outward deflection; while similar radial expansion and pressure moderation occur approaching the rotor plane, the core’s initial proximity to the spring distinguishes this regime from preceding stages.

At the Z = −5.5 mm pressure contour plane, both pre-spring and single-spring regimes exhibit identical pressure distribution patterns with high-pressure cores localized within the vortex generator’s passage; whereas the dual-spring regime demonstrates an outward shift of the high-pressure core due to flat spring deployment. Across all regimes, progressive radial expansion of high-pressure zones occurs with downstream flow development, yet all remain confined within the upstream straight-blade rotor’s flow domain. Maximum pressure magnitudes escalate from 193.94 Pa (pre-spring) to 2133.33 Pa (single-spring), culminating at 6787.88 Pa in the dual-spring regime.

**Figure 12:**
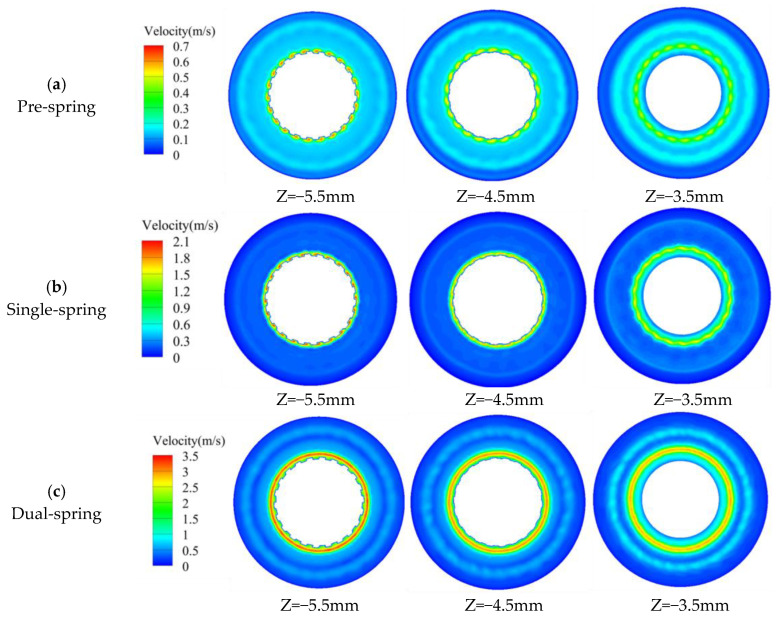
Slicing velocity cloud map of the internal structure of the angular momentum flowmeter.

Figure 12 presents velocity contour plots across three transverse sections (Z = −5.5 mm proximal to the vortex generator; Z = −3.5 mm adjacent to the upstream straight-blade rotor) of the assembly. In the pre-spring regime (Figure 12a), the high-velocity core localizes centrally within the vortex generator’s flow passage; progressing toward the upstream rotor (Z = −3.5 mm), velocity magnitude progressively moderates while the core radially diffuses. The single-spring regime (Figure 12b) maintains spatial consistency of this high-velocity core within the generator’s channel—unchanged by the undeployed flat spring—though velocity intensity attenuates and the core radially expands approaching the rotor plane. Conversely, the dual-spring regime (Figure 12c) exhibits high-velocity concentration near the outwardly deflected flat spring; similar velocity moderation and radial diffusion occur toward the rotor plane, yet the core’s spring-proximal origin distinguishes this regime from predecessors.

At the Z = −5.5 mm velocity contour plane, both pre-spring and single-spring regimes exhibit identical velocity distribution patterns with high-velocity cores confined within the vortex generator’s passage; conversely, the dual-spring regime demonstrates an outward shift of the high-velocity core due to flat spring deployment. Across all regimes, progressive radial expansion of high-velocity zones occurs during downstream flow development while remaining constrained within the upstream straight-blade rotor’s flow domain. Maximum velocity magnitudes increase from 0.68 m/s (pre-spring) to 2.13 m/s (single-spring), culminating at 3.49 m/s in the dual-spring regime.

**Figure 13:**
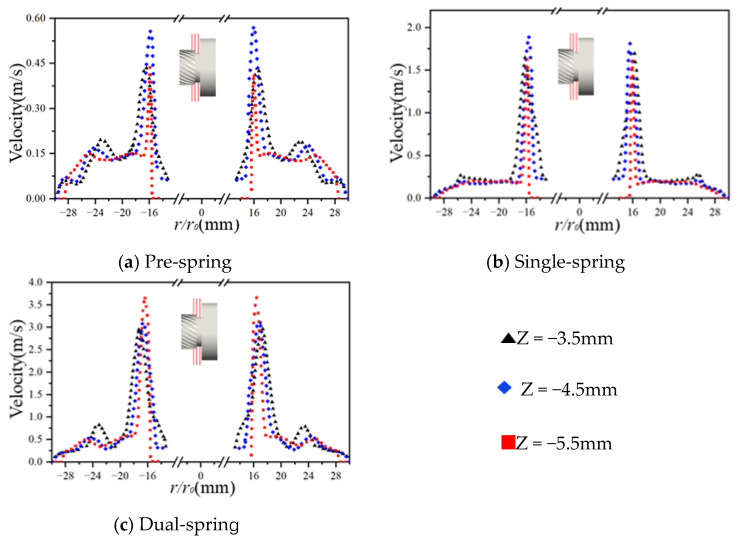
Radial velocity distribution of the outlet section slice of the cyclone.

Figure 13 depicts radial velocity distributions within the constant-diameter inter-component region between the vortex generator and upstream straight-blade rotor, with radial position (X/Y axes) as the independent variable. Maximum velocities occur at Z = −4.5 mm for both pre-spring and single-spring regimes, while shifting to Z = −5.5 mm in the dual-spring regime. Notably, all three regimes exhibit bimodal velocity profiles characterized by primary and secondary peaks; correlating with Figure 6, the secondary peak likely originates from recirculation vortices within the clearance between the vortex generator’s flat spring and housing.

**Figure 14:**
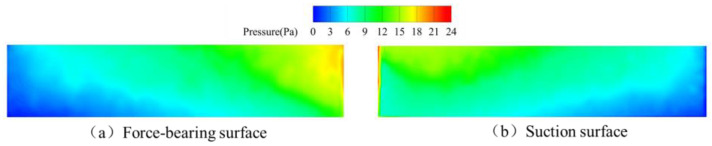
Pressure distribution of upstream rotor blades during the pre-spring response stage.

The complex internal flow characteristics within the angular momentum flowmeter arise not only from stationary flow-path components but are primarily attributable to fluid disturbances induced by the rotational effects of the upstream straight-blade rotor. Figure 14 depicts pressure distributions across pressure and suction surfaces of a single blade during the pre-spring regime: On the pressure surface, higher pressure occurs near the leading edge due to significant fluid impingement, progressively diminishing toward the trailing edge where flow separation creates a low-pressure zone. Conversely, the suction surface exhibits high pressure at the leading edge that gradually decreases downstream. At this low-flow regime, inadequate flow momentum prevents full blade coverage, resulting in trailing-edge flow separation and consequent low-pressure zone formation.

**Figure 15:**
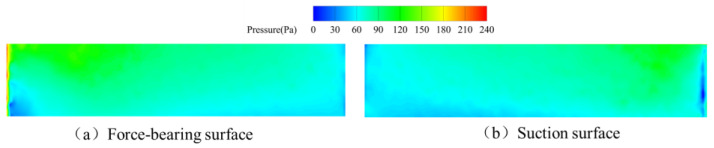
Pressure distribution of upstream rotor blades during the single-spring response stage.

As depicted in Figure 15, pressure distributions across pressure and suction surfaces of a single upstream straight-blade rotor blade during the single-spring regime reveal distinct characteristics: On the pressure surface, a low-pressure zone forms at the leading edge while uniform pressure prevails across the remaining chord—indicating complete fluid momentum transfer over the entire surface. Conversely, the suction surface exhibits high pressure in the upper-mid region of the leading edge, juxtaposed with low pressure near the blade root, likely attributable to vortex interactions generated by the vortex generator; pressure gradients progressively attenuate along the streamwise direction. This regime’s elevated flow velocity ensures comprehensive blade coverage, resulting in stabilized pressure distributions beyond the leading-edge regions where localized pressure differentials persist.

**Figure 16:**
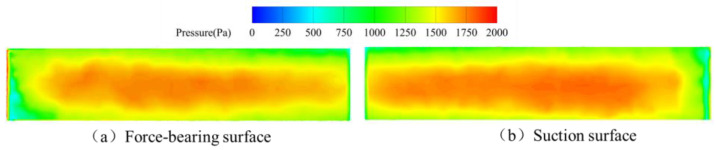
Pressure distribution of upstream rotor blades during the dual-spring response stage.

Pressure distributions across pressure and suction surfaces of a single upstream straight-blade rotor blade during the dual-spring regime (Figure 16) reveal: On the pressure surface, a low-pressure zone forms at the leading edge while uniform pressure dominates the mid-chord region—indicating comprehensive fluid momentum transfer over the blade’s primary portion. Conversely, the suction surface exhibits high pressure in the upper-mid leading-edge section juxtaposed with low pressure near the blade root, likely attributable to vortex structures generated by the vortex generator; pressure gradients progressively stabilize downstream. This regime’s further increased flow velocity ensures effective coverage across the pressure surface’s critical areas, resulting in stabilized pressure distributions beyond the leading-edge anomalies where localized pressure differentials persist.

Comparative analysis of blade pressure distributions across all three response regimes reveals a consistent pressure ridge at the suction surface leading edge, accompanied by a low-pressure zone near the blade root. The pressure ridge likely results from impingement of unconfined swirling fluid at the blade inlet, while the root-region low pressure originates from vortex structures generated by the vortex generator—per Bernoulli’s principle, accelerated local flow reduces static pressure. In both single-spring (Figure 15) and dual-spring regimes (Figure 16), a low-pressure zone forms at the pressure surface leading edge. This phenomenon can be attributed to vortex development at increased flow rates; Bernoulli’s principle similarly dictates that flow acceleration reduces static pressure, establishing this characteristic low-pressure region.

## 5. Conclusions

This study investigates the dynamic responses of internal components and flow characteristics within an angular momentum flowmeter through integrated visualization experiments and numerical simulations. Experimental imaging captured real-time rotational dynamics of the upstream straight-blade rotor and angular deflection of the downstream rotor. Numerically, transient flow behavior was resolved using a sliding mesh technique coupled with angular momentum balance theory, enabling automated iterative computation of rotor kinematics to characterize intrinsic flow mechanisms.

Through visualization experiments of the angular momentum flowmeter, it was observed that the upstream straight-bladed rotor initiates rotation at a flow velocity of 0.041 m/s, while the spiral spring coupled with the downstream straight-bladed rotor begins to respond at a flow velocity of 0.428 m/s with an initial deflection of 10°. Three distinct operational regimes were quantitatively identified. A critical transition to the dual-spring state, characterized by the response of the flat spring, occurs at a flow velocity of 0.856 m/s. Beyond this threshold, the rotational speed of the upstream straight-bladed rotor stabilizes within a narrow range of 242–250 rpm.

Numerical simulations demonstrate strong agreement with visualization data: As flow rates increase sufficiently to deploy the flat spring, the rotational speed of the upstream straight-blade rotor ceases to escalate and stabilizes within a defined range. Vortex evolution mechanisms fundamentally differ across regimes: Pre-spring and single-spring stages exhibit dual-vortex structures within the flat spring-housing clearance, with the secondary vortex undergoing significant spatial displacement—positioned in the upper-right quadrant relative to the primary vortex during the pre-spring stage, then migrating to the lower-left quadrant in the single-spring stage. This migration directly correlates with momentum redistribution driven by increased circumferential velocity in the primary vortex. In the dual-spring regime, kinetic energy intensification within the primary vortex dissipates the secondary vortex, yielding a singular dominant structure. The flat spring’s deployment modulates fluid kinetic-to-pressure energy conversion by altering passage geometry. The single-spring regime facilitates low-flow operation by enabling rotor response at minimal flow rates, extending the flowmeter’s lower measurement limit; conversely, the high-pressure/high-velocity dual-spring regime enhances stability under large-flow conditions. Flow characteristics around the upstream rotor are governed by synergistic interactions among vortex generator-induced swirl, velocity stratification, and boundary layer effects. Pre-spring stages suffer pressure non-uniformity due to insufficient flow momentum, while single/dual-spring regimes achieve improved impact coverage at higher flows—albeit with localized pressure fluctuations induced by vortex expansion.

## Figures and Tables

**Table 1:** Comparison of Fluent Rotating Structure Calculation Methods.

	Name	Moving Reference Frame	Dynamic Mesh	Sliding Mesh
Feature				
Time form		Steady state	Transient	Transient
Form of movement		Simple rotation/translation	Arbitrary deformation/fluid–structure coupling	Complex rotation/translation
Computational efficiency		High (second-level convergence)	Low (Sky level)	Medium (hourly level)
Grid processing		Do not deform	Deformation or reconstruction	Sliding without deformation

**Table 2:** Number of grid cells in angular momentum flowmeter [1 * 10^6^].

Title 1	Set1	Set2	Set3	Set4	Set5
Number of elements	3.47	4.78	5.25	6.89	7.92

**Table 3:** Dynamic response of key components of the angular momentum flowmeter.

Flow [kg/s]	Velocity [m/s]	Flat Spring Opening Degree [°]	Rotor Speed [rpm]	The Deflection Angle of the Spiral Spring [°]
0.050	0. 027	--	--	--
0.062	0.041	--	9.98	--
0.091	0.121	--	41.9	--
0.104	0.428	--	197.4	13.20
0.208	0.856	2.8	242	30.64
0.315	1.296	3.9	250	46.40
0.401	1.645	4.7	250.3	58.92
0.504	2.074	5.4	249.9	74.24
0.601	2.469	6.3	242.2	88.38
0.718	2.954	6.9	250	105.76
0.810	3.313	7.6	242	119.31

## Data Availability

The original contributions presented in this study are included in the article. Further inquiries can be directed to the corresponding author.
